# Clinical efficacy of plasmid encoding p62/SQSTM1 (Elenagen) in combination with gemcitabine in patients with platinum-resistant ovarian cancer: a randomized controlled trial

**DOI:** 10.3389/fonc.2024.1343023

**Published:** 2024-02-12

**Authors:** Sergei Krasny, Yauheni Baranau, Sergey Polyakov, Ekaterina Zharkova, Olga Streltsova, Aliona Filimonava, Volha Siarheyeva, Sviatlana Kazlouskaya, Anton Khorau, Vladimir Gabai, Alexander Shneider

**Affiliations:** ^1^ N. N. Alexandrov National Cancer Centre of Belarus, Minsk, Belarus; ^2^ Minsk City Clinical Oncologic Centre, Minsk, Belarus; ^3^ CureLab Oncology, Inc., Boston, MA, United States; ^4^ Department of Molecular Biology, Ariel University, Ariel, Israel

**Keywords:** chemotherapy, DNA vaccine, immunotherapy, chemoresistance, platinum

## Abstract

**Background:**

The purpose of this trial is to evaluate the safety and efficacy of ELENAGEN, a novel anticancer therapeutic DNA plasmid encoding p62/SQSTM1 protein, as an adjuvant to chemotherapy with gemcitabine (GEM) in patients with advanced platinum-resistant ovarian cancer.

**Methods:**

This open-label prospective randomized study with two arms. GEM (1000 mg/m^2^) on days 1 and 8 every 3 weeks was administered in both arms: in the Chemo arm (n = 20), GEM was the only treatment, and in the ELENAGEN arm (n = 20), GEM was supplemented with ELENAGEN (2.5 mg i.m. weekly). The primary endpoint was progression-free survival (PFS), and the secondary endpoint was safety. Antitumor activity was assessed by RECIST 1.1, and criteria safety was assessed according to NCI CTCAE version 5.0.

**Results:**

According to the cutoff data, the median follow-up was 13.8 months. There were no serious adverse events related to ELENAGEN treatment. The median PFS was 2.8 and 7.2 months in the Chemo and ELENAGEN arms, respectively (p Log-Rank = 0.03). Notably, at the time of cutoff, 9 patients (45%) in the ELENAGEN arm did not progress, with the longest PFS recorded thus far being 24 months. Subgroup analysis of patients in both arms demonstrated high efficacy of ELENAGEN in patients with worse prognostic factors: high pretreatment levels of CA125 and progression after platinum-free interval <3 months.

**Conclusions:**

The addition of ELENAGEN to gemcitabine is effective in patients with platinum-resistant ovarian cancer, including those with a worse prognosis.

**Clinical trial registration:**

https://www.clinicaltrials.gov/study/NCT05979298, identifier NCT05979298, 2023-08-07.

## Background

Approximately 20 000 new cases of ovarian cancer (OC) are diagnosed in the US every year, and its overall 5-year survival rate is about 50% (1). This high lethality occurs because patients are mainly diagnosed with OC at later stages, and, following front-line therapy, tumors eventually become chemoresistant (2). Combination of platinum-based chemotherapy with taxanes still remains the standard of care for advanced and recurrent OC, but recurrent OC remains difficult to treat due to chemotherapy resistance (2). Despite introduction of antiangiogenic and poly ADP-ribose polymerase I (PARP) inhibitors in recent years, they only modestly improved patient’s progression-free survival (3–5). Thus, novel OC therapeutics to improve long-term outcomes are urgently needed.

Recently, immunotherapy of cancer, especially with immune-checkpoint inhibitors (ICI), emerged as a novel treatment option for a number of solid tumors, and it was also tested in several clinical trials with OC (6). However, unlike other tumor types, the results of these trials were not encouraging. For instance, in patients with platinum-resistant OC, compared with standard chemotherapy with gemcitabine (GEM) or pegylated liposomal doxorubicin (PLD), PFS with the ICI nivolumab (anti-PDL1 antibody) was only 2.0 vs 3.8 months with GEM or PLD, and OS was 10.1 vs 12.1 months (7). Additionally, grade 3-related adverse events (AEs) occurred in 33% of patients in the nivolumab group (7). In the JAVELIN Ovarian 200 phase III trial of 566 patients with platinum-resistant OC, the addition of another anti-PD-L1 antibody, avelumab, to standard PLD treatment did not significantly increase PFS (3.7 vs 3.5 months) or OS (15.7 vs 13.1 months) (8). Furthermore, serious treatment-related adverse events occurred in 18% of patients in the combination group, compared with 11% in the PLD-only group (8). Thus, at present, the application of ICIs in the treatment of platinum-resistant OC does not appear encouraging.

We have recently developed a novel anticancer therapeutic, ELENAGEN, based on plasmid DNA encoding the p62 (SQSTM1) protein (9). p62 is a multifunctional protein that participates in selective autophagy, signal transduction, the inflammatory response and other processes (10). p62 can be a good target for anticancer vaccines since its levels are elevated in almost all human tumors tested thus far, and it increases when tumors progress (see ref ( (11, 12) for review). While p62 is dispensable for normal cells, tumors require p62 for growth and metastasis (11). Importantly, p62 levels are also increased in OC and are associated with poor prognosis and platinum resistance, making p62 a good target for the immune response elicited by ELENAGEN (13, 14).

We conducted a preclinical study of the antitumor activity of ELENAGEN on several types of solid tumors in rodents. The drug showed its effectiveness on four types of solid tumors in mice (breast carcinoma, lung carcinoma, melanoma and sarcoma) as well as breast carcinoma in rats. Importantly, we observed suppression of metastasis in three different mouse models (9). Additionally, we conducted a pilot study of Elenagen in dogs with spontaneous mammary tumors, which are much closer to human breast tumors than transplantable tumors in rodents. We found that Elenagen in dogs exerted its effects in two ways: 1) in neoadjuvant settings, it made invasive and nonresectable tumors resectable, and 2) if mastectomy was impossible, tumors completely stopped growing during the period of observation (15, 16). Importantly, no toxicity of ELENAGEN was observed in either rodents or dogs (9, 15, 16).

Furthermore, we conducted a phase I/IIa clinical trial of ELENAGEN used as a monotherapy (17). In that study, ELENAGEN showed promise in treating patients with advanced disease for which all standard methods of treatment were exhausted. For example, the progression of OC was stopped for three or more months in 4 out of 6 patients. Importantly, in contrast to ICI (see above), AEs during ELENAGEN treatment were only Grade 1, and no severe AEs were observed (17). These data encouraged us to conduct a current clinical study of ELENAGEN with platinum-resistant OC.

In addition to evoking antitumor T- and B-cell immune responses (9, 15, 16), ELENAGEN can also alleviate chronic inflammation by suppressing the generation of proinflammatory cytokines such as TNF, IL-1, and IL-6 in different rodent disease models (18, 19). In contrast to acute inflammation, which is beneficial for the immune response to microbes and cancer cells, intratumoral chronic inflammation is detrimental since it disables immune cells, thus suppressing antitumor immunity (see ref (20) for review). Since most chemotherapeutics (at least partially) engage the immune system as part of their antitumoral mechanism of action (21), chronic inflammation decreases sensitivity to chemotherapy and prevents drug delivery to tumors (22), and alleviation of chronic inflammation can enhance the effect of chemotherapy.

Therefore, two mechanisms of ELENAGEN action, as an anticancer vaccine and anti-inflammatory drug, are complimentary and can make it a unique anticancer therapeutic in combination with chemotherapeutic agents for the treatment of OC.

## Patients and methods

### Study design and patients

This single-country open-label prospective randomized two-center study with two arms was performed from January 2020 until August 2022.

Eligible patients were ≥18 years old; had measurable ovarian cancer per RECIST 1.1 criterion that had progressed <6 months after completion of platinum-based therapy; had an Eastern Cooperative Oncology Group performance status (ECOG PS) of 0 or 1; and had adequate hematologic and organ functions.

The patients were randomly assigned in a 1:1 ratio. Forty patients underwent randomization, 20 were assigned to receive chemotherapy alone (GEM) 1000 mg/m^2^ days 1,8 every 3 weeks) and 20 were assigned to receive the same chemotherapy supplemented with ELENAGEN (2.5 mg i.m. weekly).

The primary end point was progression-free survival as assessed by investigators.

The secondary endpoints were overall response rate and safety.

According to the data cutoff, the median follow-up was 13.8 months.

### Assessment and endpoints

In the safety analysis set and in the efficacy-evaluable set, all patients who received ≥ 1 dose (20 patients in each arm) were included. Safety was assessed on the basis of adverse events (AEs) and serious AEs (SAEs) according to NCI Common Terminology Criteria for Adverse Events version 5.0.

Antitumor activity was assessed by the investigator according to RECIST 1.1 criteria. Evaluation of the therapeutic effect was carried out by computer tomography (CT) every 9 weeks 19-20 days after each 3rd course of chemotherapy (before the 4th, 7th, and 10th courses, on a visit for follow-up and completion of treatment, and, if necessary, on unscheduled visits).

### Statistical analyses

Tumor response was evaluated according to the RECIST criteria ver. 1.1. PFS was defined as the time from randomization to objective disease progression on imaging or death from any cause and was assessed using the Kaplan−Meier method. PFS in the two treatment arms was compared using an unstratified two-sided log-rank test. A P < 0.05 was considered statistically significant. For the subgroup analyses, a proportional Cox regression model was used.

## Results

### Patient characteristics

**Figure 1:**
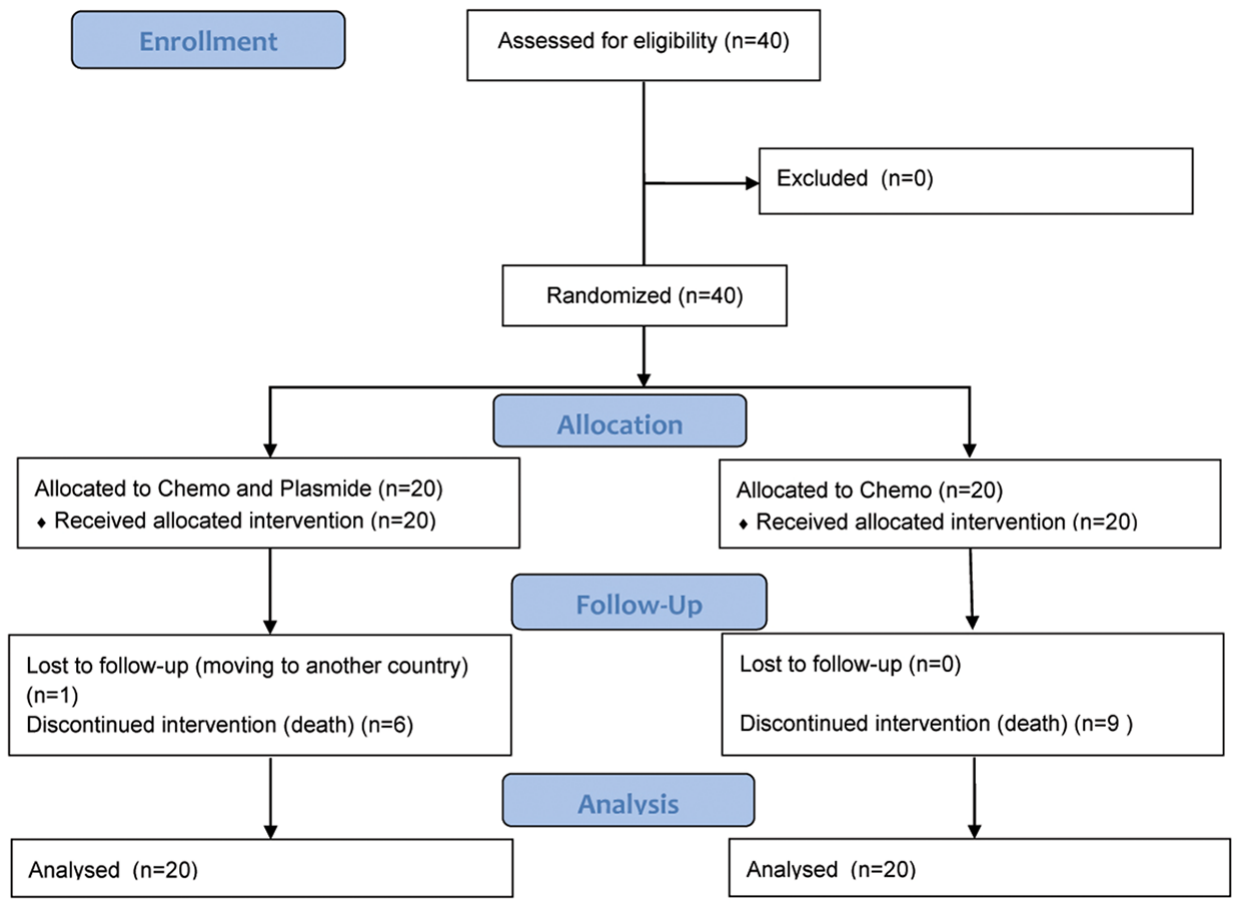
Flow diagram of patients included in the analysis.

Patient characteristics are summarized in **Table 1**. The most common histological type of platinum-resistant OC in both groups was high-grade serous adenocarcinoma. More than half of the patients in both groups progressed after only one line of platinum-based chemotherapy with platinum-free intervals of 3-6 months. Additionally, the majority of patients in both groups had high levels of CA125 as well as metastases in the peritoneum (75-85%) and elsewhere (**Table 1**). **Figure 1** represents flow diagram of PROC patients included in the analysis

**Table 1:** Baseline Patient Characteristics.

Characteristic	Chemo		ELENAGEN	
	No	%	No	%
Age, years				
Median	54.6		54.2	
Range	33.6-65.5		32.8-69.6	
ECOG PS				
0	14	70	13	65
1	6	30	7	35
Histology at diagnosis				
Serous/adenocarcinoma	17	85	15	75
Clear cell	2	10	3	15
Adenocarcinoma	1	5	1	5
Mucinous	0	0	1	5
Histologic grade at diagnosis				
1	3	15	1	5
2	1	5	0	0
3	15	75	19	95
No data	1	5	0	0
Platinum-free interval				
Up to 3 months	7	35	8	40
3-6 months	13	65	12	60
No line of chemo for platinum sensitive ovarian cancer				
1	11	55	12	60
2	5	25	7	35
3	4	20	1	5
CA125				
Normal	5	25	4	20
High	15	75	16	80
Metastatic lesions				
Peritoneum	15	75	17	85
Peritoneal effusion	9	45	7	35
Lymph nodes	8	40	15	75
Liver	4	20	6	30
Lung	3	15	4	20
Pleural effusion	1	5	3	15
Soft tissue	5	25	3	15
Spleen	0	0	0	0
Bone	1	5	1	5

### Safety

Safety was assessed in all 40 patients. During the study period, one death was registered in the ELENAGEN arm without any evidence of disease progression within 2 months after randomization, and its possible cause was venous embolism. Although autopsy was not performed and the final diagnosis was not determined, this adverse event was counted as thrombosis and unrelated to the disease. One patient in the ELENAGEN arm underwent surgery due to intestinal obstruction within one month after randomization, and the subsequent cycle of the treatment was delayed for three weeks. After recovery from the surgery, the patient continued treatment without evidence of progression to the cutoff date (up to 19 months).

The majority of adverse events in the GEM and ELENAGEN arms were caused by GEM and were presented by different types of hematological toxicity. No cases of febrile neutropenia or other life-threatening complications that required hospitalization occurred. The cases of intestinal obstruction and metabolic toxicity were caused by organ compression by gross tumor mass. Only skin rash, itching and redness at the injection site were considered to be related to ELENAGEN administration. At the same time, the number of adverse events with grade <= 3 and AEs of special interest (potentially related to plasmid administration) did not significantly differ between the groups (**Table 2**).

**Table 2:** Adverse events Grade <= 3 and of special interest.

Adverse event	Chemo arm		ELENAGEN arm	
	No	%	No	%
Neutropenia	4	20	7	35
Thrombocytopenia	2	10	4	20
Anemia	1	5	2	10
ALT/AST increase	1	5	0	0
Creatinine increase	1	5	0	0
Thrombosis	1	5	1	5
Intestinal obstruction	0	0	1	5
AE of special interest				
Skin rash G1	0	0	2	10
Itching G1	0	0	2	10

A slight increase in the number of hematological adverse events in the ELENAGEN arm was apparently related to the longer GEM exposure due to increased PFS.

### Efficacy

The tumor response was assessed according to the RECIST 1.1 criteria. No complete responses were observed in either group. The objective response rate was higher in the ELENAGEN arm: partial response (PR) 5.9% and 26.7%, stable disease (SD) 35.3% and 53.3%, and disease progression 58.8% and 20.0% in the Chemo and ELENAGEN arms, respectively. In total, the disease control rate (PR and SD) was significantly higher in the ELENAGEN arm (80.0% vs 41.2% in the Chemo and ELENAGEN arms, respectively, p = 0,001). One patient in the ELENAGEN arm was able to undergo complete cytoreduction with no evidence of disease progression.

**Figure 2:**
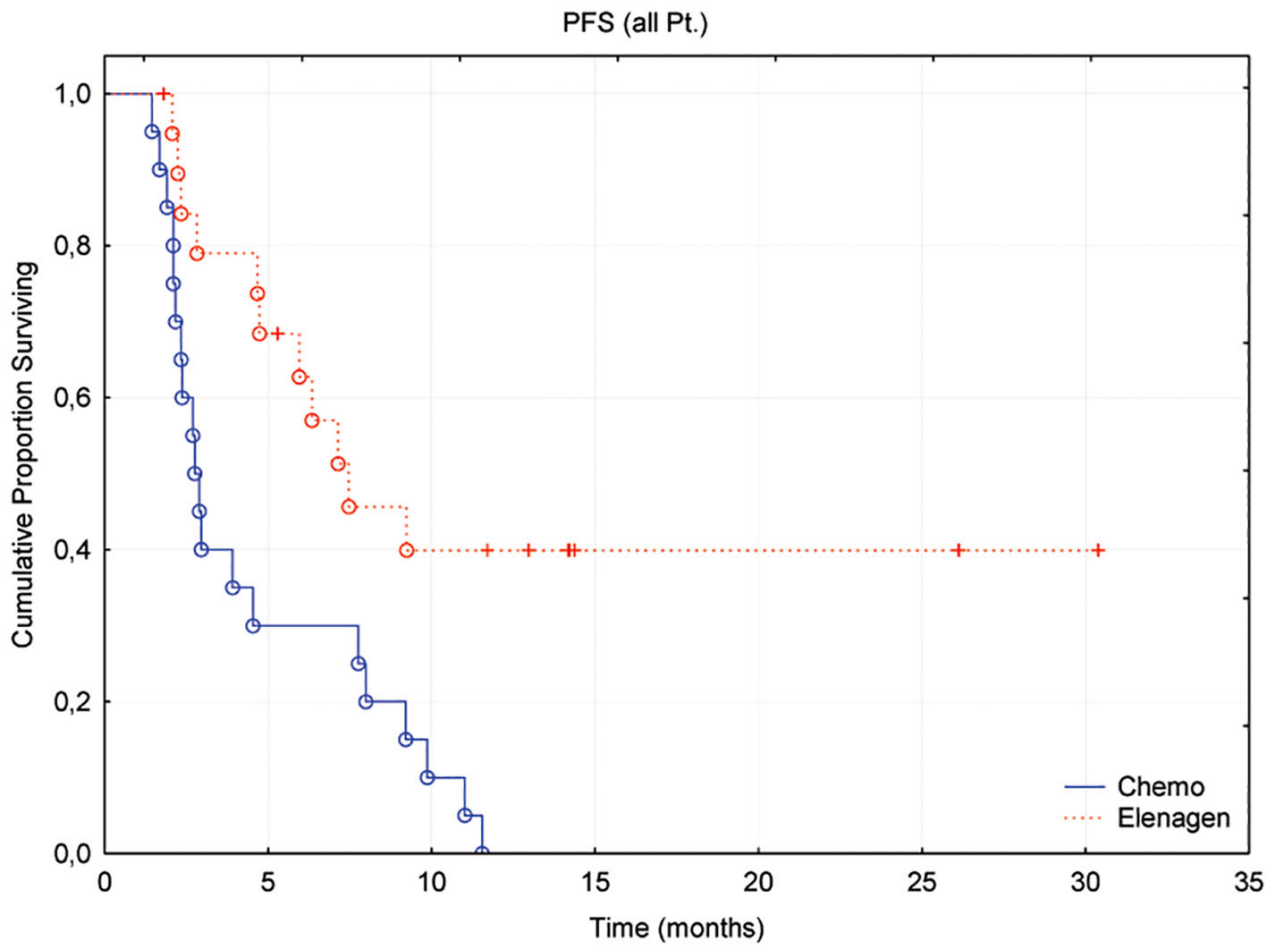
Progression-free survival of patients treated with Chemo+ELENAGEN or Chemo only.

The median progression-free survival (PFS) was 2.8 and 7.2 months in the Chemo and ELENAGEN arms, respectively (p Log-Rank = 0.03) (**Figure 2**). For the lower 25^th^ percentile (lower quartile), these numbers were 2.1 vs. 4.2 months, respectively, while for the upper quartile (75^th^ percentile), 7.7 months, it was only possible to determine for the chemotherapy group alone.

Notably, at the time of cutoff, 9 patients (45%) in the ELENAGEN arm did not progress, with the longest PFS recorded thus far being 24 months.

### Subgroup analysis

We assessed the efficacy of ELENAGEN in subgroups with different basic characteristics.

The peritoneal effusion, CA125 level (normal or high), platinum-free interval (PFI), (up to 3 months vs 3-6 months), number of treatment lines for platinum-sensitive ovarian cancer and histological type of tumor (serous vs non-serous) were chosen as potential predictive factors. Cox proportional hazards regression analyses were performed (**Table 3**).

**Table 3:** COX regression model.

	Hazard ratio	95% CI	P Value
Peritoneal effusion	0,8	0.3 – 2.1	0,622
CA125 Level (normal vs high)	10,8	2.4 – 48.3	**0,002**
PRFI (up to 3 vs 3-6 months)	1,4	1.0 – 2.0	**0,039**
Number lines of Chemo for PSOC	1,0	0.5 – 1.9	0,889
Histology (serous vs nonserous)	1,7	1.1 – 2.6	**0,022**

PRFI, platinum-resistance free interval.

PSOC, platinum-sensitive ovarian cancer.

Bold p values are statistically significant.

The CA125 level (normal or high), platinum-free interval (up to 3 months vs 3-6 months) and histological type of tumor (serous vs non-serous) were statistically significant in the Cox model.

**Figure 3:**
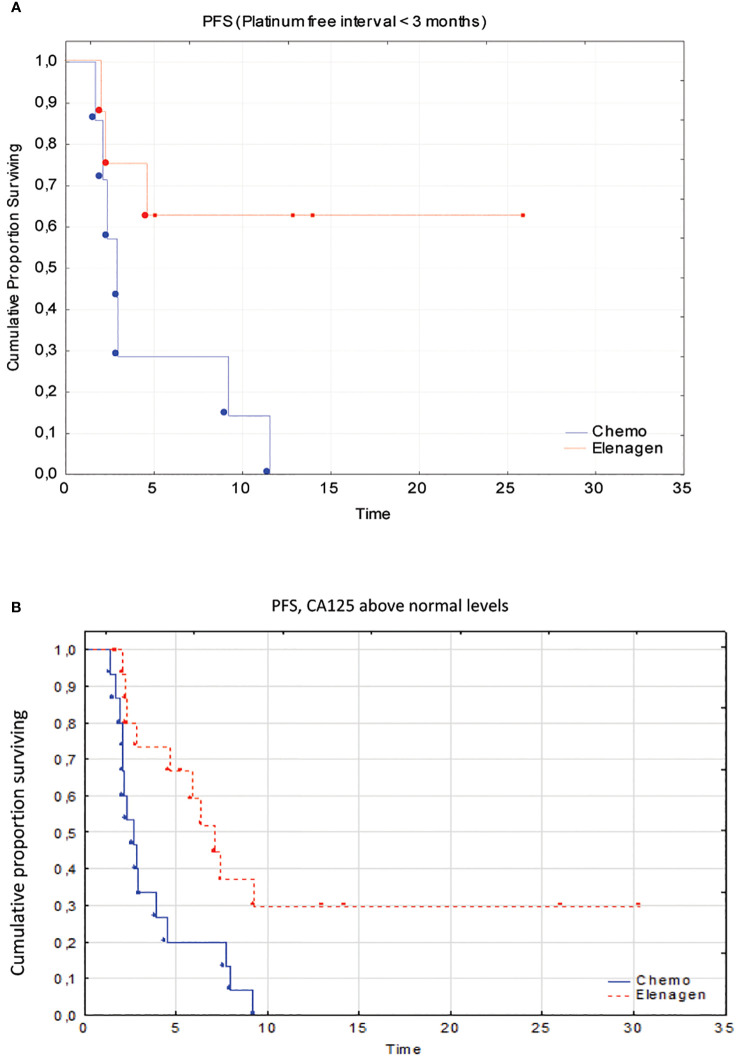
Subgroup analysis of patients with a platinum-free interval <3 months **(A)** and above normal CA125 levels **(B)**.

However, due to the low number of patients with non-serous cancer (n=5 in both groups), additional analysis for histological type was not performed, but we performed pairwise comparisons of PFS in the Chemo and ELENAGEN arms according to the identified prognostic factors CA 125 level and PFI. The initial high CA-125 level and short PFI significantly affected PFS (**Table 4**; **Figure 3**).

**Table 4:** Progression-free survival (PFS) in subgroups.

Subgroups	`Median PFS (months)		p Log-Rank
	Chemo	ELENAGEN	
CA125 high level	2.5 (2.1-4.1)	6.5 (2.7-NR)	p Log-Rank = 0.01
PRFI up to 3 months	2.6 (2.0-4.5)	NR	p Log-Rank = 0.03
PRFI 3-6 months	2.7 (2.1-7.0)	6.7 (4.3-NR)	p Log-Rank = 0.05

PRFI, platinum-resistance free interval.

## Discussion

Platinum-resistant OC, even if treated with a standard therapy such as gemcitabine, PLD, paclitaxel, and topotecan, has a dismal prognosis: a medium PFS of 3-4 months and an OS of 12 months (23, 24). Therefore, a more effective therapy for this form of OC is urgently needed. Despite the success of immunotherapy with immune checkpoint inhibitors (ICIs) in some tumors (25)), such a combination of ICIs with chemotherapy in OC has not yet been successful, and this treatment was quite toxic (6, 8) (see Background). Thus, at present, the application of ICIs in the treatment of platinum-resistant OC does not appear encouraging.

Our study demonstrated that the addition of our novel plasmid drug ELENAGEN to a standard chemotherapy regimen with GEM had a profound effect on PFS, increasing it from 2.8 months to 7.2 months. Importantly, no signs of increased toxicity of this combined treatment compared to GEM alone were found. Remarkably, ELENAGEN in combination with GEM was also effective in patients with a dismal prognosis: progression after platinum therapy within 3 months and with high pretreatment levels of CA125. For instance, a recent meta-analysis of data from more than 10 000 patients demonstrated that the increased serum level of CA-125 before treatment correlated with poor progression-free survival (HR=1.59, 95% CI=1.44~1.76, p<0.001) and overall survival (HR=1.62, 95% CI=1.270-2.060, p<0.001) (26). We are aware that due to a low number of patients in our subgroup analysis, these observations should be evaluated in larger trials.

ELENAGEN operates through at least two complementary mechanisms. First, ELENAGEN can work as an immunotherapeutic by activating T- and B-cellular antitumor immune responses by inducing the generation of antibodies and T-lymphocytes to p62 (9, 16) and stimulating the accumulation of T-lymphocytes in tumors (15). Since OC, especially platinum-resistant OC, has higher levels of p62 than normal tissue (13, 14, 27, 28), such an immune response to p62 may contribute to the antitumor activity of ELENAGEN. Furthermore, it is reasonable to combine elenagen with chemotherapy since anticancer drugs are currently believed to engage, at least partially, the immune system (see ref (21) for review), which may increase the antitumor activity of ELENAGEN. Indeed, the combination of chemotherapy with ICI immunotherapy in some tumors had a greater effect than either treatment alone, and such combinations are approved by the FDA (25). Accordingly, in our previous study, we found that patients with breast and ovarian cancers achieved additional tumor stabilization for 3-7 months when subjected to chemotherapy following ELENAGEN treatment even if the tumors were initially chemoresistant (17, 29).

Second, ELENAGEN was shown to decrease chronic inflammation (30), which may hamper the effect of chemotherapy (22). Elevated levels of the proinflammatory cytokine IL-6 in the serum or ascites of OC patients correlated with chemoresistance, particularly platinum resistance (31), and higher ascites levels of IL-6 and TNF predict worse PFS in patients with OC (32). Thus, decreasing chronic inflammation ELENAGEN may promote the effect of chemotherapy in OC. Last but not least, in dogs with mammary tumors, we found that ELENAGEN treatment results in tumor shrinkage, changes in the structure of the tumor matrix and lowering the grade of the tumors (15, 16). Such tumor “normalization” may also contribute to sensitization to chemotherapy. Finally, Elenagen treatment dramatically changes the expression of collagen isoforms (16), making it easier for tumor-infiltrating lymphocytes (TILs) to enter the tumor and harder for metastatic cells to exit. Thus, these effects of elenagen make it a unique anticancer therapeutic.

In conclusion, the addition of ELENAGEN to gemcitabine is effective in patients with ovarian cancer, including those with a worse prognosis. Future studies of ELENAGEN with various tumors and chemotherapy regimens are warranted.

## Data availability statement

All data generated or analyzed during this study are included in this Article. Data not shown in the manuscript are available from the corresponding author on reasonable request.

## Ethics statement

The study was approved by the Ministry of Health of Belarus (#03-19) and ethical review boards of N. Alexandrov National Cancer Centre of Belarus and Minsk City Cancer Center. Informed consent were signed by all study participants.

## Author contributions

SKr: Conceptualization, Data curation, Funding acquisition, Investigation, Project administration, Resources, Supervision, Writing – review & editing. YB: Conceptualization, Data curation, Formal Analysis, Investigation, Methodology, Project administration, Software, Writing – review & editing. SP: Funding acquisition, Project administration, Resources, Supervision, Writing – review & editing. EZ: Data curation, Formal Analysis, Validation, Writing – review & editing. OS: Investigation, Methodology, Project administration, Resources, Writing – review & editing. AF: Formal Analysis, Investigation, Resources, Writing – review & editing. VS: Investigation, Methodology, Writing – review & editing. SKa: Investigation, Software, Validation, Writing – review & editing. AK: Data curation, Investigation, Methodology, Project administration, Writing – review & editing. VG: Conceptualization, Data curation, Formal Analysis, Funding acquisition, Methodology, Resources, Writing – original draft, Writing – review & editing. AS: Conceptualization, Supervision, Resources, Writing – review & editing.
